# Temporomandibular disorders in a tertiary clinic: associations with pain, chronicity, sleep versus awake bruxism, and psychological factors—a retrospective study

**DOI:** 10.22514/jofph.2026.010

**Published:** 2026-01-12

**Authors:** Thaviporn Limrachtamorn

**Affiliations:** ^1^Faculty of Dentistry, Thammasat University, 12120 Pathum Thani, Thailand

**Keywords:** Temporomandibular disorders, Orofacial pain, Bruxism, Psychological factors, Clinical characteristics, Retrospective study

## Abstract

**Background**: Temporomandibular disorders (TMD) are prevalent orofacial pain conditions; however, the interrelationships among clinical, psychological, and behavioral factors remain insufficiently explored, particularly within the Thai population. This single-center retrospective study aimed to examine the associations among clinical characteristics, pain, bruxism, and psychological factors in patients with TMD. **Methods**: The medical records of 222 adult patients diagnosed with TMD at the Orofacial Pain Clinic between January and December 2024 were reviewed. The primary outcomes were the associations of pain severity and symptom duration with psychological factors, and the secondary outcomes were the relationships of sleep and awake bruxism with psychological factors and related variables. Statistical analyses included nonparametric tests, chi-square tests, and logistic regression. **Results**: Among the 222 patients (75.7% female; mean age 35.83 ± 17.08 years), 56.8% presented with chronic symptoms, 36.0% reported sleep bruxism, and 37.8% reported awake bruxism. Higher pain severity was significantly associated with depression (*p* = 0.004), anxiety (*p* = 0.001), and stress (*p* = 0.045). Chronic symptoms (>3 months) were associated with depression, anxiety, and stress (*p * < 0.001). Awake bruxism demonstrated significant associations with all three psychological factors (*p * < 0.001), whereas sleep bruxism did not show such associations. In multivariable analyses, patients with acute symptoms (≤3 months) had lower odds of sleep bruxism compared with those with chronic symptoms (odds ratio (OR) = 0.37, *p* = 0.003), and higher stress levels were associated with awake bruxism (OR = 1.15, *p * < 0.001). **Conclusions**: The findings highlight the burden of psychological factors among TMD patients, particularly those with higher pain intensity, chronic symptoms, and awake bruxism. Awake bruxism may serve as a clinical indicator of psychological factors, underscoring the importance of psychological screening and biopsychosocially oriented management. Nonetheless, the results should be interpreted with caution given the retrospective design and the single-center setting.

## 1. Introduction

Temporomandibular disorders (TMD) comprise a group of dysfunctions involving the temporomandibular joint (TMJ), masticatory muscles, and related structures [1]. They represent one of the most frequent causes of orofacial pain of non-odontogenic origin, meaning pain not arising from the teeth [2]. The most common symptoms reported by TMD patients include pain in the masticatory muscles or preauricular region, which often worsens with chewing or jaw movement. In addition to pain, patients frequently experience limited jaw movement and TMJ noises [3]. According to the Diagnostic Criteria for TMD (DC/TMD) taxonomy, TMDs are currently classified into four major subgroups: temporomandibular joint disorders (*e.g.*, arthralgia, disc displacement, and degenerative joint disease), masticatory muscle disorders (*e.g.*, myalgia and spasm), headache attributed to TMD, and disorders of associated structures (*e.g.*, coronoid hyperplasia) [4].

Epidemiological data indicates that TMDs are more prevalent in females than in males, and a recent meta-analysis reported a female-to-male ratio of approximately 1.75:1, with an overall prevalence of 29.5% in the general population. Interestingly, the prevalence is higher in individuals younger than 18 years (38.5%) than in those aged 18 years or older (34.1%) [5]. The prevalence of painful TMD, however, is lower, with a pooled estimate of 26.4% [6]. Pain associated with TMD has been linked to substantial impairment in daily functioning and quality of life, often manifesting as mood disturbances and heightened anxiety [7].

The pathogenesis of TMD is considered multifactorial and may be best explained through the biopsychosocial model, which integrates biological, psychological, and social dimensions [8, 9], with predisposing factors including impaired general health, psychological factors, and parafunctional habits such as bruxism. Initiating factors most often involve trauma or excessive mechanical loading of the masticatory system, while perpetuating factors may be behavioral (*e.g.*, clenching, grinding, or abnormal head posture), social (*e.g.*, pain perception or learned pain behaviors), or emotional (*e.g.*, depression and anxiety). A single factor may simultaneously contribute to multiple domains; for instance, bruxism may act as a predisposing, initiating, and perpetuating factor [10, 11, 12].

Bruxism is defined as repetitive jaw-muscle activity involving clenching or grinding of the teeth, or bracing or thrusting of the mandible. It is classified as either sleep bruxism or awake bruxism. The 2018 international consensus proposed three diagnostic certainty levels: possible bruxism, based on self-report only; probable bruxism, based on clinical inspection with or without self-report; and definite bruxism, confirmed by instrumental assessment, such as polysomnography (PSG) for sleep bruxism or electromyography (EMG) for awake bruxism, with or without clinical or self-reported findings [13]. The updated 2025 international consensus recommended replacing these hierarchical categories with terminology that directly reflects the assessment methods employed: subject-based (self-report), clinically based (clinical examination), and device-based (instrumental assessment) [14]. Bruxism has been implicated in increased masticatory muscle loading, microtrauma, and inflammation, thereby exacerbating TMD symptoms [15].

Psychological factors such as anxiety, depression, stress, and somatization have been shown to be closely associated with both the development and progression of TMD. Patients with TMD consistently demonstrate higher levels of anxiety and depression compared with healthy controls, supporting the view that psychological factors contribute significantly to its pathophysiology [16, 17]. Stress, in particular, may increase muscle tone in the cranio-cervical region and promote parafunctional behaviors such as clenching or bruxism. Moreover, heightened sympathetic nervous system activity under stress can further augment muscle tension, thereby creating conditions that predispose to pain in the masticatory and cervical muscles. Stress-induced sympathetic hyperactivity has, therefore, been proposed as a key mechanism that exacerbates TMD symptoms [18].

Nevertheless, only a limited number of studies, involving patients diagnosed according to the DC/TMD, have directly compared sleep and awake bruxism in relation to both psychological and clinical factors, and such evidence remains particularly scarce in the Thai population. To address this gap, the present study was conducted as a single-center retrospective observational analysis aimed at examining the associations among clinical characteristics, pain intensity, symptom duration, types of bruxism, and psychological factors in adult patients with TMD. Given its retrospective design and reliance on clinically based assessment of bruxism, the findings should be regarded as associative rather than causal. It was hypothesized that bruxism, particularly awake bruxism, would be associated with higher levels of depression, anxiety, and perceived stress.

## 2. Materials and methods

### 2.1 Study design and participants

This single-center retrospective observational study was conducted using medical records of consecutive new patients who attended the Orofacial Pain Clinic, a tertiary care facility at the Faculty of Dentistry, Thammasat University, between 01 January and 31 December 2024. The study protocol was reviewed and approved by the Human Research Ethics Committee of Thammasat University (Science), Thailand (Certificate of Exemption No. 014/2568; approval code 68DE032). All procedures adhered to the principles of the Declaration of Helsinki, the Belmont Report, and the International Conference on Harmonisation-Good Clinical Practice (ICH-GCP) guidelines. The requirement for informed consent was waived by the Committee because data were obtained exclusively from fully de-identified medical records before analysis. All eligible patient charts were reviewed by the author as part of a quality assurance process. The study followed the Strengthening the Reporting of Observational Studies in Epidemiology (STROBE) guidelines for observational research, and a participant flow diagram (Fig. 1) illustrates the numbers screened, eligible, and excluded, together with reasons for exclusion. The dataset was complete with no missing data. All analyses were performed according to a prespecified analysis plan defined before data extraction. Since this study was exploratory, no a priori power analysis was performed.

**Fig. 1.** STROBE-compliant participant flow diagram showing the number of patients screened, excluded (with reasons), and included in the final analysis. TMJ: temporomandibular joint.

The inclusion criteria were: (1) first-time patients presenting to the clinic, (2) age 18 years or older, and (3) a diagnosis of TMD established according to the DC/TMD. Exclusion criteria were: (1) incomplete medical records, (2) a prior history of TMJ surgery, and (3) a history of head and neck cancer. Medical records meeting all inclusion criteria and none of the exclusion criteria were retrieved from the clinic database. The dataset included demographic variables (age and sex), chief complaints, pain intensity, and symptom duration. Psychological status was assessed using the Patient Health Questionnaire-9 (PHQ-9), the Generalized Anxiety Disorder-7 (GAD-7), and the 10-item Perceived Stress Scale (PSS-10). Clinical examination findings were also extracted, including the presence and type of bruxism, categorized as sleep bruxism or awake bruxism, as well as the final clinical diagnoses.

### 2.2 Data collection and examiners

Clinical data were initially recorded for routine patient care and extracted for research purposes. All examinations and data entry were conducted by a single staff specialist in orofacial pain who had received formal training in the DC/TMD protocol. For diagnostic consistency, the examiner underwent calibration with standard reference cases, before the study period, as part of the department’s routine quality assurance procedures. Intra-examiner reliability was evaluated in a pilot group of 20 cases, yielding Cohen’s *κ* values of ≥ 0.80 across all major diagnostic categories, thereby confirming substantial reliability. As the examinations were performed during routine clinical care, the examiner was not blinded to the study objectives. Data extraction from medical records was carried out by the same examiner and systematically verified against the original source charts to ensure accuracy. To maintain confidentiality, all personal identifiers were removed before analysis.

### 2.3 Measures

#### 2.3.1 Pain intensity

Pain intensity was evaluated using the Numerical Rating Scale (NRS), in which patients rated their current pain on a scale from 0 to 10, with 0 indicating no pain and 10 indicating the worst imaginable pain. The validated Thai version of the NRS was employed for this study [19]. In the primary analyses, NRS scores were treated as a continuous variable to maximize statistical power and sensitivity. For descriptive purposes, however, pain levels were also categorized according to the International Classification of Diseases, 11th Revision (ICD-11), as proposed by the International Association for the Study of Pain (IASP) Task Force [20]: mild (1–3), moderate (4–6), and severe (7–10). Both continuous and categorical formats were incorporated into the analyses to allow estimation of effect sizes, correlation testing, and group comparisons.

#### 2.3.2 Symptom duration

Symptom duration was determined from the patient-reported onset of TMD symptoms. Duration was initially recorded in months as a continuous variable. For categorical analyses, it was classified as acute (≤3 months) or chronic (>3 months), following the IASP definition of chronic pain [21]. Both continuous and categorical forms of this variable were retained in the analyses to facilitate the interpretability of results.

#### 2.3.3 Psychological measures

In the primary analyses, psychological instruments were examined as continuous variables (raw scores) to preserve statistical power and sensitivity. For descriptive reporting, however, scores were additionally categorized into severity levels, with detailed cut-off values provided in **Supplementary Table 1**. All instruments used in this study were validated Thai versions, and the questionnaires were self-administered by patients during their initial clinic visit.

#### 2.3.3.1 Depression

Depressive symptoms were evaluated using the PHQ-9, a widely used 9-item self-report instrument designed to screen for and assess the severity of depression [22]. The Thai validation conducted by Lotrakul *et al*. [23] demonstrated acceptable reliability, with an internal consistency coefficient (Cronbach’s alpha) of 0.79.

#### 2.3.3.2 Anxiety

Anxiety symptoms were assessed using the GAD-7 scale [24]. The Thai validation by Musumari *et al*. [25] reported high internal consistency, with a Cronbach’s alpha of 0.89.

#### 2.3.3.3 Perceived stress

Perceived stress was measured using the 10-item PSS-10 [26]. The Thai validation conducted by Wongpakaran and Wongpakaran had good reliability, with an internal consistency coefficient (Cronbach’s alpha) of 0.85 [27].

#### 2.3.4 Clinical examination findings

Clinical examination was performed following the standardized DC/TMD clinical examination protocol, and all assessments were conducted by a single examiner who was formally trained and calibrated in the protocol. The evaluation comprised several diagnostic parameters. Restricted mouth opening was defined as a maximum assisted opening of less than 40 mm, measured in millimeters with overbite correction using a ruler. TMJ noises, including clicking and crepitus, were assessed by digital palpation during mandibular movements. Muscle pain with palpation was examined by applying standardized pressure of 1 kg to the masseter and temporalis muscles, as specified in the DC/TMD protocol and confirmed during examiner calibration. TMJ pain with palpation was assessed by applying a standardized pressure of 0.5 kg to the TMJs, also in accordance with the DC/TMD protocol and examiner calibration.

#### 2.3.5 Clinical diagnoses

Clinical diagnoses were established following the DC/TMD and were assigned during the initial patient visit through the use of standardized diagnostic procedures. Each diagnosis was derived from defined combinations of patient-reported symptoms and clinical examination findings, as specified in the DC/TMD protocol. Since magnetic resonance imaging (MRI) was not routinely performed in this setting, the diagnostic process was based on validated clinical criteria, which have been shown to provide acceptable diagnostic accuracy in both clinical practice and research applications [4]. The patients were permitted to receive one or more diagnoses, encompassing the following categories: myalgia, arthralgia, headache attributed to TMD, disc displacement with reduction, disc displacement with reduction with intermittent locking, disc displacement without reduction with limited opening, disc displacement without reduction without limited opening, degenerative joint disease, and subluxation.

#### 2.3.6 Types of bruxism

Bruxism was classified as present or absent and further divided into sleep bruxism and/or awake bruxism, since both types may occur in the same patient. The classification followed the most recent international consensus of Verhoeff *et al*. [14] (2025) and was based on a clinically based assessment. The diagnosis relied primarily on observable clinical signs, including tooth wear facets, indentations on the tongue or cheek, and hypertrophy of the masseter muscle. Self-reported information recorded during the initial visit was also reviewed as supportive evidence. Patients were asked about habitual tooth clenching or grinding during wakefulness or sleep and whether these behaviors had been observed or reported by a bed partner. This combined approach was selected to provide a balance between diagnostic reliability and feasibility in the context of a retrospective study, in which instrumental (device-based) assessments were not available.

### 2.4 Statistical analysis

All statistical analyses were performed using IBM SPSS Statistics, version 26.0 (IBM Corp., Armonk, NY, USA). Descriptive statistics were calculated for all variables. Continuous variables were summarized as mean ± standard deviation (SD) when distributions were approximately normal, or as median and interquartile range (IQR) when distributions were non-normal; ranges were additionally reported where relevant. Categorical variables were expressed as frequencies and percentages with corresponding 95% confidence intervals (CIs), calculated using the Wilson score method. All statistical tests were two-sided, with significance defined as *p * < 0.05 and *q* < 0.05 for false discovery rate (FDR)—adjusted analyses. Normality was assessed with the Shapiro-Wilk test, where *p * < 0.05 was considered evidence of non-normality, and nonparametric methods were applied when assumptions of normality were not met.

The Kruskal-Wallis test was used to examine differences in psychological assessment scores (PHQ-9, GAD-7, PSS-10) across pain severity groups. When omnibus results were significant, pairwise Mann-Whitney U tests with Bonferroni correction (α/3) were conducted. Effect sizes were reported as *η*^2^(*H*) for Kruskal-Wallis, *r* for Mann-Whitney U, and Cramér’s *V *for chi-square analyses. Associations between bruxism and psychological factors, as well as between bruxism and muscle or TMJ pain, were analyzed using chi-square tests. Comparisons of psychological scores between acute and chronic symptom groups were performed with the Mann-Whitney U test, and associations between pain intensity (NRS) and psychological measures were examined using Spearman’s rank correlation.

Separate multivariable logistic regression models were constructed for sleep bruxism and awake bruxism. Independent variables included psychological scores (entered as continuous covariates), age, sex, pain intensity, and symptom chronicity. Model fit was assessed using −2 log likelihood, Nagelkerke *R*^2^, the Hosmer-Lemeshow goodness-of-fit test, and classification accuracy. The results are reported as odds ratios (ORs) with 95% CIs.

Multiplicity across hypothesis families was controlled using procedures appropriate to each analysis: Holm adjustment for omnibus Kruskal-Wallis tests and acute—chronic comparisons, Bonferroni correction for *post hoc* Mann-Whitney U tests, and FDR adjustment (*q* < 0.05) for chi-square analyses of bruxism and psychological strata. For logistic regression models, no multiplicity correction was applied across coefficients; instead, interpretation focused on effect sizes and confidence intervals.

Although no a priori power calculation was performed, *post hoc* evaluation indicated that the sample size (N = 222) provided adequate precision for prevalence estimates, with 95% confidence interval half-widths of ±6–7%. The sample also ensured events-per-variable ratios greater than 10 for logistic regression models and approximately 80% power to detect small-to-moderate effects (*r* ≈ 0.25–0.30; |*ρ*| ≥ 0.20). Due to limited power, interaction analyses were treated as exploratory.

## 3. Results

### 3.1 Demographic characteristics and chief complaints

A total of 222 patients were included, of whom 75.7% were female. The median age was 29 years, with a mean of 35.83 ± 17.08 years. Jaw pain was the most frequently reported chief complaint, followed by preauricular pain and joint noise. The demographic characteristics and chief complaints are presented in Table 1.

**Table 1.** Demographic characteristics, symptom duration, and chief complaints of the study participants (N = 222).

Variables		N (%), Mean ± SD, or Median (IQR)	95% CI
Demographic characteristics			
	Total patients	222	–
Gender			
	Male	54 (24.3%)	19.15–30.37%
	Female	168 (75.7%)	69.63–80.85%
Age (yr)			
	Mean ± SD	35.83 ± 17.08	–
	Median (IQR)	29.00 (22.00–49.00)	–
	Range	18–88	–
Symptom duration (mon)			
	Mean ± SD	13.67 ± 23.68	–
	Median (IQR)	4.00 (1.00–12.00)	–
	Range	0.03–132.00	–
Symptom duration (categorical)			
	Acute (≤3 mon)	96 (43.2%)	36.90–49.82%
	Chronic (>3 mon)	126 (56.8%)	50.18–63.10%
Chief complaints			
	Jaw pain	106 (47.7%)	41.27–54.30%
	Preauricular pain	36 (16.2%)	11.95–21.63%
	Joint noise	26 (11.7%)	8.12–16.61%
	Sleep bruxism	25 (11.3%)	7.74–16.10%
	Ear pain	9 (4.1%)	2.15–7.52%
	Limited mouth opening	8 (3.6%)	1.84–6.95%
	Temple pain	4 (1.8%)	0.70–4.54%
	Jaw deviation	4 (1.8%)	0.70–4.54%
	Headache	2 (0.9%)	0.25–3.22%
	Open locking	2 (0.9%)	0.25–3.22%

*SD: standard deviation; 95% CI: 95% confidence interval (Wilson score method); IQR: interquartile range.* *Chief complaints are based on patient-reported symptoms at the initial visit, which may differ from clinically established diagnoses reported elsewhere in the manuscript.*

### 3.2 Pain intensity

The median NRS score for pain intensity was 4.00. When classified by severity, 10.4% of patients reported no pain, 29.7% reported mild pain, 41.0% reported moderate pain, and 18.9% reported severe pain. The distribution of pain severity levels with 95% confidence intervals is shown in Fig. 2.

**Fig. 2.** Distribution of patients by pain severity classification based on the NRS (N = 222). Values above the bars indicate counts with percentages, and error bars represent 95% confidence intervals for proportions, calculated using the Wilson score method. NRS: Numerical Rating Scale.

### 3.3 Symptom duration

The median symptom duration was 4.00 months. Based on the IASP definition, 43.2% of patients were classified as having acute symptoms, while 56.8% had chronic symptoms. Symptom duration data are presented in Table 1.

### 3.4 Psychological assessments

For depression, the median PHQ-9 score was 5.00, with nearly half of patients classified as having minimal depression, approximately one-third were classified as mild, and a smaller proportion reported moderate to severe symptoms. For anxiety, the median GAD-7 score was 4.50, with about half of the patients reporting minimal anxiety and progressively fewer classified as mild, moderate, or severe. For perceived stress, the median PSS-10 score was 18.50, with patients distributed across mild, moderate, and severe categories in relatively comparable proportions. The distribution of psychological assessment scores by severity level is presented in Table 2.

**Table 2.** Classification of psychological assessment scores by severity level (N = 222).

Scale	Severity level	N (%)
PHQ-9		
	Minimal depression	99 (44.6%)
	Mild depression	70 (31.5%)
	Moderate depression	39 (17.6%)
	Moderately severe depression	13 (5.9%)
	Severe depression	1 (0.5%)
GAD-7		
	Minimal anxiety	111 (50.0%)
	Mild anxiety	65 (29.3%)
	Moderate anxiety	29 (13.1%)
	Severe anxiety	17 (7.7%)
PSS-10		
	Mild stress	77 (34.7%)
	Moderate stress	66 (29.7%)
	Severe stress	79 (35.6%)

*PHQ-9: Patient Health Questionnaire-9; GAD-7: Generalized Anxiety Disorder-7; PSS-10: 10-item Perceived Stress Scale.*

### 3.5 Clinical examination findings

Restricted mouth opening was observed in 6.3% of patients, TMJ clicking was observed in 58.1%, and crepitus in 9.5%. Muscle pain upon palpation was reported by 82.9%, whereas TMJ pain upon palpation was noted in 48.2%. These clinical examination findings are summarized in Table 3.

**Table 3.** Clinical examination findings and clinical diagnoses among study participants (N = 222).

Variables		Yes (N)	Yes (%)	95% CI	No (N)	No (%)
Clinical examination findings						
	Restricted mouth opening	14	6.3	3.79–10.31%	208	93.7
	TMJ clicking	129	58.1	51.53–64.41%	93	41.9
	TMJ crepitus	21	9.5	6.27–14.03%	201	90.5
	Muscle pain with palpation	184	82.9	77.38–87.27%	38	17.1
	TMJ pain with palpation	107	48.2	41.71–54.75%	115	51.8
Clinical diagnoses						
	Myalgia	184	82.9	77.38–87.27%	38	17.1
	Arthralgia	107	48.2	41.71–54.75%	115	51.8
	Headache attributed to TMD	6	2.7	1.24–5.77%	216	97.3
	Disc displacement with reduction	115	51.8	45.25–58.29%	107	48.2
	Disc displacement with reduction with intermittent locking	14	6.3	3.79–10.31%	208	93.7
	Disc displacement without reduction with limited opening	0	0.0	0.00–1.70%	222	100.0
	Disc displacement without reduction without limited opening	0	0.0	0.00–1.70%	222	100.0
	Degenerative joint disease	21	9.5	6.27–14.03%	201	90.5
	Subluxation	13	5.9	3.45–9.76%	209	94.1

*95% CI: 95% confidence interval (Wilson score method); TMJ: temporomandibular joint; TMD: temporomandibular disorders.*

### 3.6 Clinical diagnoses

The most frequent diagnosis was myalgia, present in 82.9% of patients, followed by disc displacement with reduction in 51.8% and arthralgia in 48.2%. Less common diagnoses included degenerative joint disease (9.5%), disc displacement with reduction with intermittent locking (6.3%), subluxation (5.9%), and headache attributed to TMD (2.7%). No patients were diagnosed with disc displacement without reduction. The distribution of clinical diagnoses is presented in Table 3.

### 3.7 Types of bruxism

Sleep bruxism was identified in 80 patients (36.0%, 95% CI: 30.01–42.54%), and awake bruxism was identified in 84 patients (37.8%, 95% CI: 31.72–44.37%).

### 3.8 Types of bruxism and muscle or TMJ pain with palpation

Chi-square tests revealed no statistically significant associations between either sleep bruxism or awake bruxism and the presence of muscle pain or TMJ pain on palpation (all *p * > 0.05). For sleep bruxism, no significant association was found with muscle pain (ꭓ^2^ = 0.066, *df* = 1, *p* = 0.797) or TMJ pain (ꭓ^2^ = 0.190, *df* = 1, *p* = 0.663). Similarly, awake bruxism was not significantly associated with muscle pain (ꭓ^2^ = 2.883, *df* = 1, *p* = 0.090) or TMJ pain (ꭓ^2^ = 0.176, *df* = 1, *p* = 0.675), although a non-significant trend was observed for muscle pain. After applying Holm’s step-down procedure to control for multiple testing across the four prespecified chi-square analyses (sleep bruxism *vs*. muscle pain, sleep bruxism *vs*. TMJ pain, awake bruxism *vs*. muscle pain, and awake bruxism *vs*. TMJ pain), none of the associations reached statistical significance (sleep bruxism *vs*. muscle pain: Holm-adjusted *p* = 1.000; sleep bruxism *vs*. TMJ pain: Holm-adjusted *p* = 1.000; awake bruxism *vs*. muscle pain: Holm-adjusted *p* = 0.360; awake bruxism *vs*. TMJ pain: Holm-adjusted *p* = 1.000).

### 3.9 Pain severity and psychological assessment scores

Kruskal-Wallis tests revealed significant differences across pain severity groups for all three psychological measures, including PHQ-9 (*H* = 11.21, *p* = 0.004, *η*^2^(*H*) = 0.06), GAD-7 (*H* = 13.50, *p* = 0.001, *η*^2^(*H*) = 0.07), and PSS-10 (*H* = 6.21, *p* = 0.045, *η*^2^(*H*) = 0.03). *Post hoc* Mann-Whitney U tests with Bonferroni correction indicated that patients in the severe pain group had significantly higher psychological scores than those in the mild and moderate pain groups, whereas no significant differences were observed between the mild and moderate groups. Effect sizes (*r*) for these pairwise comparisons ranged from 0.24 to 0.38. After correction for multiple testing using Holm’s step-down procedure across the three outcomes, omnibus results remained significant (PHQ-9: Holm-adjusted *p* = 0.008; GAD-7: Holm-adjusted *p* = 0.003; PSS-10: Holm-adjusted *p* = 0.045). Pairwise comparisons of psychological assessment scores are summarized in Table 4.

**Table 4.** Pairwise comparisons of psychological assessment scores across pain severity groups.

Pain severity comparison	PHQ-9 (*p*-value)	*r*	GAD-7 (*p*-value)	*r*	PSS-10 (*p*-value)	*r*
Mild *vs*. Moderate	0.444	0.06	0.373	0.07	0.646	0.04
Moderate *vs*. Severe	0.001^a,^**	0.29	0.001^b,^**	0.29	0.016^c,^*	0.24
Mild *vs*. Severe	<0.001^d,^***	0.38	<0.001^e,^***	0.35	0.011^f,^*	0.25

*PHQ-9: Patient Health Questionnaire-9; GAD-7: Generalized Anxiety Disorder-7; PSS-10: 10-item Perceived Stress Scale; r: effect size (rank-biserial correlation).* *p*-values are based on Mann-Whitney U tests. Bonferroni-adjusted α = 0.017 was used to determine statistical significance. *p  < 0.05, **p  < 0.01, ***p  < 0.001. Superscripts denote pairwise comparisons: ^a^PHQ-9 (Moderate *vs.* Severe), ^b^GAD-7 (Moderate *vs.* Severe), ^c^PSS-10 (Moderate *vs.* Severe), ^d^PHQ-9 (Mild *vs.* Severe), ^e^GAD-7 (Mild *vs.* Severe), ^f^PSS-10 (Mild *vs.* Severe).

Pain intensity was also examined as a continuous variable. Spearman’s rank correlation analyses showed significant positive associations between NRS scores and depressive symptoms (PHQ-9; *ρ* = 0.182, *p* = 0.007; **Supplementary Fig. 1**) as well as anxiety symptoms (GAD-7; *ρ* = 0.234, *p * < 0.001; **Supplementary Fig. 2**). The correlation between pain intensity and perceived stress (PSS-10; *ρ* = 0.131, *p* = 0.052; **Supplementary Fig. 3**) did not reach statistical significance. After applying Holm’s adjustment across the three correlations, significance was maintained for NRS–PHQ-9 (Holm-adjusted *p* = 0.014) and NRS–GAD-7 (Holm-adjusted *p* = 0.003), whereas the NRS–PSS-10 correlation remained non-significant (Holm-adjusted *p* = 0.052). Boxplots illustrating the distribution of PHQ-9, GAD-7, and PSS-10 scores across pain severity levels are shown in **Supplementary Figs. 4,5,6**.

### 3.10 Symptom duration and psychological assessment scores

Patients with symptoms lasting longer than 3 months demonstrated significantly higher scores across all psychological measures, including the PHQ-9, GAD-7, and PSS-10 (all *p * < 0.001, Mann-Whitney U test; **Supplementary Figs. 7,8,9**). After correction for multiple testing using Holm’s step-down procedure across the three prespecified comparisons, all associations remained statistically significant (all Holm-adjusted *p * < 0.001). The comparisons of psychological assessment scores between patients with acute (≤3 months) and chronic (>3 months) symptom duration are presented in Table 5.

**Table 5.** Comparison of psychological assessment scores between acute (≤3 months) and chronic (>3 months) symptom duration groups.

Psychological variables	Mean rank (acute ≤3 mon)	Mean rank (chronic >3 mon)	U	*Z*	*p*-value	*r*
PHQ-9	80.10	135.42	3034	−6.397	<0.001***	0.43
GAD-7	77.56	137.36	2790	−6.930	<0.001***	0.46
PSS-10	84.69	131.93	3474	−5.433	<0.001***	0.36

*PHQ-9: Patient Health Questionnaire-9; GAD-7: Generalized Anxiety Disorder-7; PSS-10: 10-item Perceived Stress Scale; U: Mann-Whitney U statistic; *Z*: standardized test statistic; *r*: effect size (rank-biserial correlation).* *p*-values are based on Mann-Whitney U tests comparing acute (≤3 months) and chronic (>3 months) symptom duration groups. ***p  < 0.001.

### 3.11 Types of bruxism and psychological assessment scores

Sleep bruxism was not significantly associated with PHQ-9, GAD-7, or PSS-10 scores, although a non-significant trend was observed for GAD-7 (*p* = 0.058, *q* = 0.087). In contrast, awake bruxism showed significant associations with all three measures (all *p * < 0.001, *q* < 0.001), with effect sizes in the medium-to-large range (Cramér’s *V*: PHQ-9 = 0.387; GAD-7 = 0.423; PSS-10 = 0.425). The cross-tabulated counts, test statistics, and effect sizes are shown in Table 6.

**Table 6.** Association between types of bruxism and psychological assessment scores.

Types of bruxism	Psychological variable	Severity level	Bruxism n (%)	No bruxism n (%)	χ^2^	*df*	*p*-value	*q* (FDR)	Cramér’s *V*
Sleep bruxism	PHQ-9	Minimal	30 (37.5%)	69 (48.6%)	6.979	4	0.137	0.164	0.177
		Mild	32 (40.0%)	38 (26.8%)					
		Moderate	14 (17.5%)	25 (17.6%)					
		Moderately severe	3 (3.8%)	10 (7.0%)					
		Severe	1 (1.3%)	0 (0.0%)					
	GAD-7	Minimal	34 (42.5%)	77 (54.2%)	7.501	3	0.058	0.087	0.184
		Mild	32 (40.0%)	33 (23.2%)					
		Moderate	10 (12.5%)	19 (13.4%)					
		Severe	4 (5.0%)	13 (9.2%)					
	PSS-10	Mild	26 (32.5%)	51 (35.9%)	1.066	2	0.587	0.587	0.069
		Moderate	22 (27.5%)	44 (31.0%)					
		Severe	32 (40.0%)	47 (33.1%)					
Awake bruxism	PHQ-9	Minimal	22 (26.2%)	77 (55.8%)	33.329	4	<0.001***	<0.001***	0.387
		Mild	26 (31.0%)	44 (31.9%)					
		Moderate	24 (28.6%)	15 (10.9%)					
		Moderately severe	11 (13.1%)	2 (1.4%)					
		Severe	1 (1.2%)	0 (0.0%)					
	GAD-7	Minimal	24 (28.6%)	87 (63.0%)	39.766	3	<0.001***	<0.001***	0.423
		Mild	28 (33.3%)	37 (26.8%)					
		Moderate	16 (19.0%)	13 (9.4%)					
		Severe	16 (19.0%)	1 (0.7%)					
	PSS-10	Mild	11 (13.1%)	66 (47.8%)	40.170	2	<0.001***	<0.001***	0.425
		Moderate	23 (27.4%)	43 (31.2%)					
		Severe	50 (59.5%)	29 (21.0%)					

*PHQ-9: Patient Health Questionnaire-9; GAD-7: Generalized Anxiety Disorder-7; PSS-10: 10-item Perceived Stress Scale; χ^2^: chi-square statistic; *df*: degrees of freedom; *p*: two-sided *p*-value; *q*: FDR-adjusted *p*-value; Cramér’s *V*: effect size; FDR: false discovery rate. Cross-tabulated counts (n, %) are shown for each severity category.* *p*-values are based on chi-square tests with FDR adjustment. ***p  < 0.001.

### 3.12 Multivariable logistic regression analyses for sleep and awake bruxism

In adjusted logistic regression models, acute symptom duration (≤3 months) was independently associated with lower odds of sleep bruxism compared with chronic symptoms (>3 months) (OR = 0.37, 95% CI: 0.19–0.72, *p* = 0.003). None of the psychological measures, age, sex, or pain intensity were significantly associated with sleep bruxism. For awake bruxism, higher perceived stress scores (PSS-10) were independently associated with increased odds (OR = 1.15, 95% CI: 1.09–1.22, *p * < 0.001). Symptom chronicity showed a borderline association (OR = 0.50, 95% CI: 0.24–1.04, *p* = 0.064). Full regression results with model fit indices are presented in Table 7.

**Table 7.** Multivariable logistic regression models of factors associated with sleep and awake bruxism.

Covariate	Sleep bruxism OR (95% CI)	*p*-value	Awake bruxism OR (95% CI)	*p*-value
PHQ-9	1.02 (0.90–1.16)	0.711	0.89 (0.77–1.02)	0.086
GAD-7	0.90 (0.79–1.03)	0.137	1.07 (0.93–1.24)	0.340
PSS-10	1.03 (0.98–1.08)	0.266	1.15 (1.09–1.22)	<0.001***
Pain intensity (NRS)	0.98 (0.86–1.12)	0.781	1.01 (0.87–1.18)	0.853
Symptom duration (acute ≤3 mon *vs*. chronic >3 mon)	0.37 (0.19–0.72)	0.003**	0.50 (0.24–1.04)	0.064
Age	0.99 (0.97–1.01)	0.180	1.01 (0.99–1.03)	0.330
Sex (male *vs.* female)	0.74 (0.37–1.47)	0.383	1.58 (0.72–3.46)	0.257

OR: odds ratio; 95% CI: 95% confidence interval; PHQ-9: Patient Health Questionnaire-9; GAD-7: Generalized Anxiety Disorder-7; PSS-10: 10-item Perceived Stress Scale; NRS: Numerical Rating Scale. Statistical significance was set at *p * < 0.05. **p  < 0.01, ***p  < 0.001. *Model fit indices:* *Sleep bruxism—Hosmer-Lemeshow χ^2^ = 6.28, *df* = 8, *p* = 0.62; Nagelkerke *R*^2^ = 0.09; −2 log likelihood = 274.95; overall classification accuracy = 65.3%.* *Awake bruxism—Hosmer-Lemeshow χ^2^ = 2.48, *df* = 8, *p* = 0.96; Nagelkerke *R*^2^ = 0.37; −2 log likelihood = 224.16; overall classification accuracy = 74.8%.*

## 4. Discussion

This study demonstrated that most participants were female, consistent with previous reports suggesting that hormonal influences, psychosocial factors, and heightened pain sensitivity may contribute to the greater prevalence of TMD in women [5, 28, 29, 30, 31]. The mean age of the cohort was within the adult range, and jaw pain was the most frequently reported chief complaint. Moderate to severe pain was common, and more than half of the patients experienced symptoms persisting for longer than 3 months. Psychological factors were also prevalent, with approximately one-third of the sample exhibiting moderate to severe levels of depression, anxiety, or stress. In terms of clinical diagnoses, myalgia was the most frequently observed, followed by disc displacement with reduction and arthralgia, consistent with findings from meta-analytic evidence [5]. Both sleep and awake bruxism were frequently identified, with awake bruxism reported in 37.8% of patients and sleep bruxism in 36.0%. Although these estimates were derived from a TMD population, the pattern aligns with a recent meta-analysis that found a higher global prevalence of awake bruxism (23%) compared with sleep bruxism (21%) [32].

Regarding the association between bruxism and pain characteristics, this study found no statistically significant relationships between either sleep or awake bruxism and muscle or TMJ pain with palpation. Although bruxism has been associated with muscle tenderness, pain, and TMD through mechanisms of musculoskeletal overloading, recent investigations suggest that this relationship cannot be explained by a simple linear model. Orofacial pain conditions are increasingly understood as multifactorial, arising from the interplay of biological, psychological, and behavioral components, which may help explain the absence of significant associations in this cohort [33]. Consistent with this, a systematic review reported weaker associations in studies employing PSG or EMG compared with those based on self-reported diagnoses [34]. In the present study, a non-significant trend between awake bruxism and masticatory muscle pain (*p* = 0.090) suggests a possible association. This observation aligns with prior evidence indicating a higher prevalence of awake bruxism in patients with TMD [35] and its positive association with TMD signs and symptoms [36]. Similarly, a population-based study found no significant association between sleep bruxism and TMD symptoms, while awake bruxism demonstrated clear associations [37]. Taken together, the lack of significant associations in the current study may reflect the reliance on clinically based bruxism assessment without device-based confirmation, the greater complexity of patients in a tertiary care setting, and the multifactorial nature of TMD pain, in which bruxism represents only one of several contributing factors.

Patients with chronic symptoms exhibited significantly higher PHQ-9, GAD-7, and PSS-10 scores, and pain severity was positively correlated with psychological factors. This observation is consistent with prior evidence showing greater somatic symptom burden, depression, and anxiety in chronic TMD pain compared with acute pain [38, 39], reflecting the bidirectional associations between TMD and psychological factors [40, 41]. Previous research has also demonstrated that psychological factors are linked to greater pain severity, increased frequency of pain episodes, and prolonged symptom duration, likely mediated through mechanisms of central sensitization [16, 42, 43]. Conceptual models further suggest that chronic pain and negative emotions are intrinsically related through shared regulatory pathways, including rumination and emotional avoidance, while sustained nociceptive input with central sensitization may perpetuate a vicious cycle between pain and psychological burden [41, 43, 44, 45]. Moreover, a recent systematic review identified psychological factors as major contributors to the development of TMD [46]. Collectively, these findings underscore the importance of considering symptom chronicity when evaluating the psychological impact of TMD.

Awake bruxism was significantly associated with higher levels of depression, anxiety, and stress, whereas sleep bruxism showed no such associations. This result is consistent with the findings of Manfredini and Lobbezoo [47], who reported that awake bruxism, particularly clenching-type activity, is strongly related to psychosocial factors and mood disturbances. Previous studies have similarly identified psychosocial variables, including anxiety, stress, and alexithymia, as important determinants of awake bruxism [48, 49, 50]. In the present study, multivariable analyses demonstrated that symptom chronicity was independently associated with sleep bruxism, whereas perceived stress was the only factor independently associated with awake bruxism after adjustment for covariates. These findings suggest that the associations between awake bruxism and psychological measures may be influenced by pain chronicity and overall symptom burden rather than representing a direct causal effect. They further support the view that sleep and awake bruxism should be considered distinct conditions with different pathophysiological mechanisms, reinforcing the role of psychological assessment in patients with bruxism [47, 51, 52]. Although the observed association between awake bruxism and psychological factors is consistent with the biopsychosocial model, its demonstration in a Thai tertiary care population highlights the clinical importance of integrating routine psychological screening into TMD assessment. Taken together, these observations emphasize the relevance of biopsychosocially oriented management strategies and underscore the need for early identification of psychological burden in awake bruxers to optimize treatment outcomes.

In contrast, sleep bruxism appears to be primarily regulated by central nervous system arousal mechanisms, with serotonergic and gamma-aminobutyric acid-ergic (GABAergic) modulation playing important roles [53]. Evidence from recent systematic reviews indicates that sleep bruxism is more common in patients with obstructive sleep apnea (OSA), potentially due to shared mechanisms, such as autonomic arousals and neurotransmitter dysregulation [54]. Polysomnographic studies have also reported associations with alterations in sleep architecture, including a possible reduction in deep sleep [55]. Despite these physiological pathways, the role of psychological factors in sleep bruxism remains uncertain. Although some studies have suggested associations [56, 57], PSG-based investigations provide little support for such relationships. For example, regardless of whether sleep bruxism was diagnosed by self-report, clinical inspection, or PSG, no significant associations with stress or anxiety were found [58], and even individuals with severe PSG-confirmed bruxism did not show greater levels of psychological factors [59]. Collectively, the absence of significant associations between sleep bruxism and psychological factors observed in this study should be interpreted cautiously, as it may reflect both methodological limitations and the predominant influence of neurophysiological mechanisms.

The findings are also consistent with the biopsychosocial model, which emphasizes the interaction of biological, psychological, and behavioral factors in the pathogenesis of TMD. Given the significant associations observed between psychological factors, pain severity, and awake bruxism, early recognition of psychological factors may improve diagnostic accuracy and support timely referral to mental health professionals. Incorporating psychological screening into routine clinical assessments may further strengthen diagnostic precision. In addition, multimodal management strategies that integrate both physical and psychological components, such as behavioral therapy, stress management, and physical rehabilitation, have the potential to improve treatment outcomes and enhance overall quality of life.

This study has several limitations. First, its retrospective observational design precludes causal inference; the associations observed, such as those between pain severity, chronicity, and psychological factors, are likely bidirectional and should be interpreted as correlations. Second, pain intensity was assessed at a single timepoint using the NRS, which, although validated in Thai, may not adequately capture temporal fluctuations. Third, bruxism classification relied on clinical inspection supplemented by self-report, without PSG or EMG confirmation, which may have led to misclassification and biased prevalence estimates and associations. Fourth, MRI was not performed in all cases, potentially reducing diagnostic accuracy and contributing to the absence of disc displacement without reduction in this cohort. MRI is not routinely indicated for all TMD patients, and its systematic use would have imposed a substantial financial burden. Fifth, subgroup analyses were not feasible because of small sample sizes. Sixth, the single-center design in a tertiary clinic with a predominantly female population limits generalizability, as the sample may reflect more severe and complex cases than those typically seen in primary care. Seventh, no a priori sample size calculation was performed. Although *post hoc *evaluation indicated adequate precision for small-to-moderate effects, some non-significant findings may reflect limited statistical power. In addition, the reliance on bivariate tests without full adjustment for confounders increases the likelihood of chance findings. Finally, potential confounding factors, including medication use, sleep quality, and systemic conditions, were not controlled and may have influenced the associations observed.

Future research could address these limitations by adopting prospective longitudinal designs and incorporating objective diagnostic tools, such as PSG for sleep bruxism and EMG for awake bruxism, to enable device-based assessment. Moreover, clinical trials targeting stress management in awake bruxism are warranted to clarify potential mechanisms and to provide evidence for standardized biopsychosocial management strategies in TMD.

## 5. Conclusions

This study, conducted in a Thai tertiary care population diagnosed with DC/TMD, shows that psychological factors are common in TMD, especially in patients with greater pain intensity, chronic symptoms, and awake bruxism. Sleep bruxism was not significantly associated with psychological measures, but this result should be interpreted cautiously because of methodological limitations. Awake bruxism, by contrast, was linked to higher levels of depression, anxiety, and stress, suggesting that it may serve as a clinical indicator of psychological factors in TMD. Routine psychological assessment, particularly in patients with awake bruxism, may improve diagnostic accuracy, guide management, and help prevent chronicity through early biopsychosocial interventions. However, because the study was retrospective, single-center, and relied on clinical rather than device-based bruxism assessment, the findings should be generalized with caution.

## Supplementary Material

Supplementary material associated with this article can be found, in the online version, at https://files.jofph.com/ files/article/2010588187091451904/attachment/ Supplementary%20material.docx.


