# Supplementary material for: Temporomandibular disorders in a tertiary clinic: associations with pain, chronicity, sleep versus awake bruxism, and psychological factors—a retrospective study
Source: J Oral Facial Pain Headache. 2026 Jan 12;40(1):106–18. doi: 10.22514/jofph.2026.010 (PMC12853168; doi:10.22514/jofph.2026.010)
Supplement: Supplementary file 1 [file Supplementary-material.docx]

Supplementary material

Supplementary Table 1. Severity levels and score ranges for PHQ-9, GAD-7, and PSS-10.

| Instrument | Score range | Severity levels |
| --- | --- | --- |
| PHQ-9 | 0–27 | 0–4 minimal, 5–9 mild, 10–14 moderate, 15–19 moderately severe, 20–27 severe |
| GAD-7 | 0–21 | 0–4 minimal, 5–9 mild, 10–14 moderate, 15–21 severe |
| PSS-10 | 0–40 | 0–13 low, 14–26 moderate, 27–40 high |

Note. PHQ-9: Patient Health Questionnaire-9; GAD-7: Generalized Anxiety Disorder-7; PSS-10: 10-item Perceived Stress Scale.


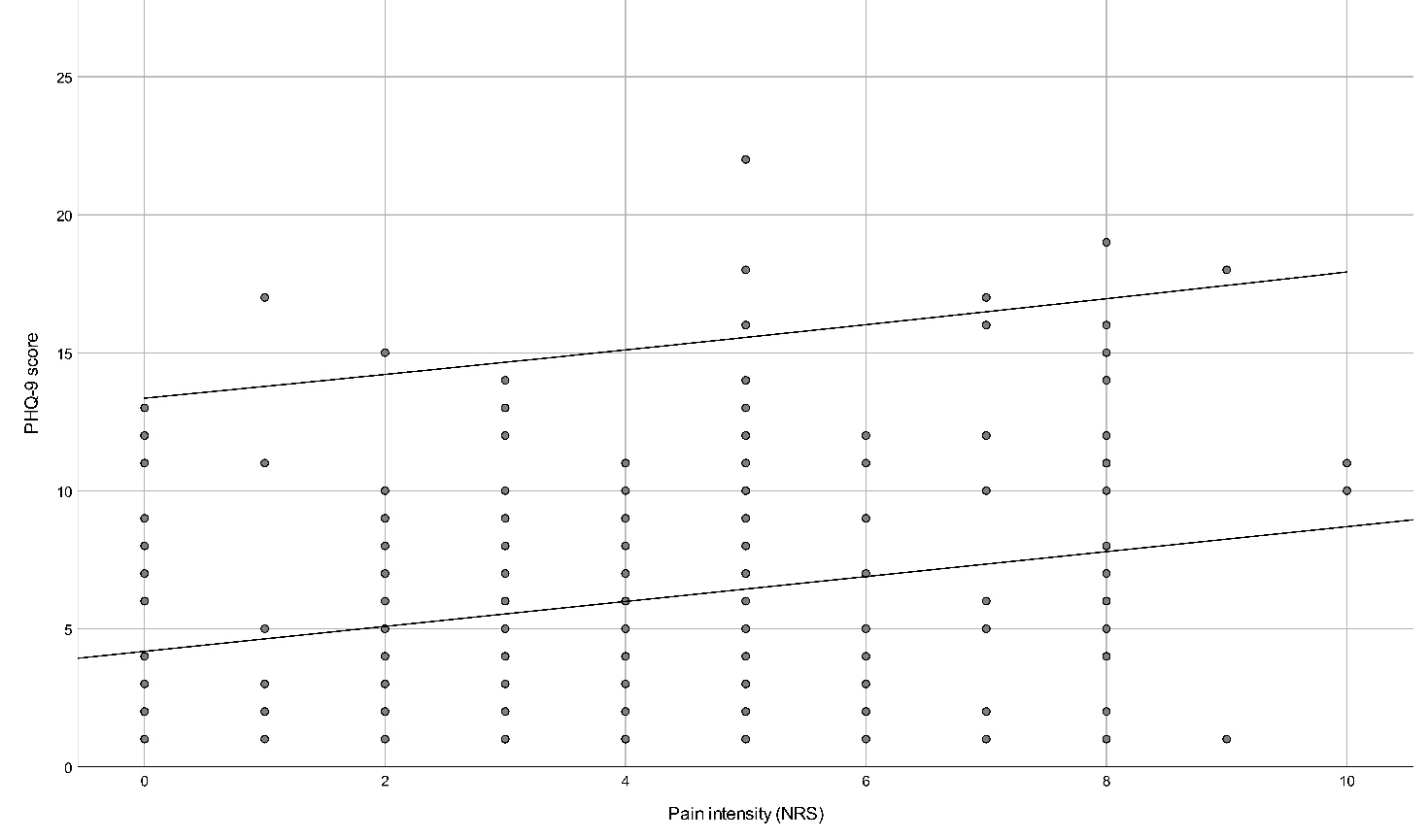


Supplementary Fig. 1. Scatterplot showing the association between pain intensity (NRS) and PHQ-9 scores. PHQ-9: Patient Health Questionnaire-9; NRS: Numerical Rating Scale.


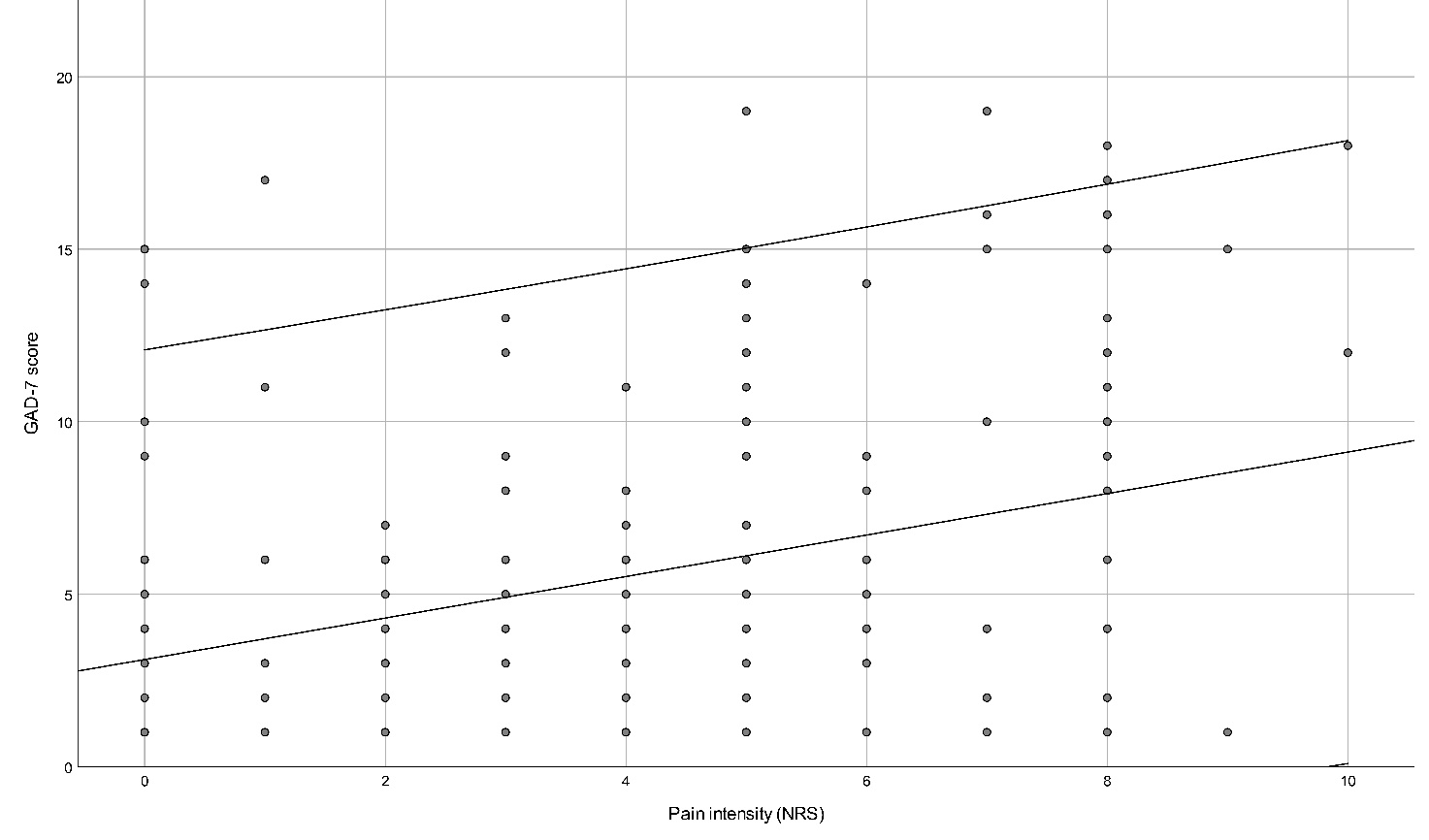


Supplementary Fig. 2. Scatterplot showing the association between pain intensity (NRS) and GAD-7 scores. GAD-7: Generalized Anxiety Disorder-7; NRS: Numerical Rating Scale.


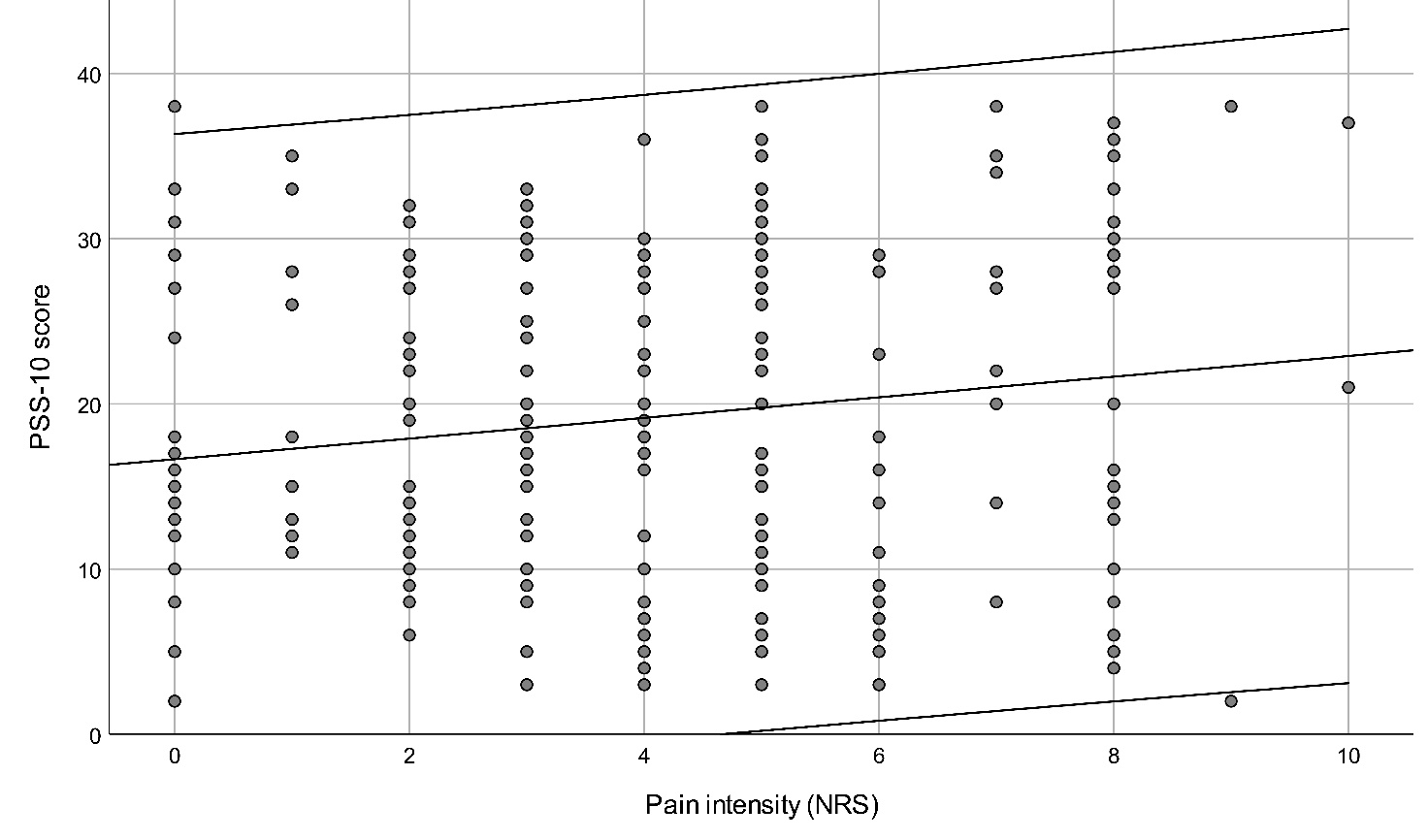


Supplementary Fig. 3. Scatterplot showing the association between pain intensity (NRS) and PSS-10 scores. PSS-10: 10-item Perceived Stress Scale; NRS: Numerical Rating Scale.


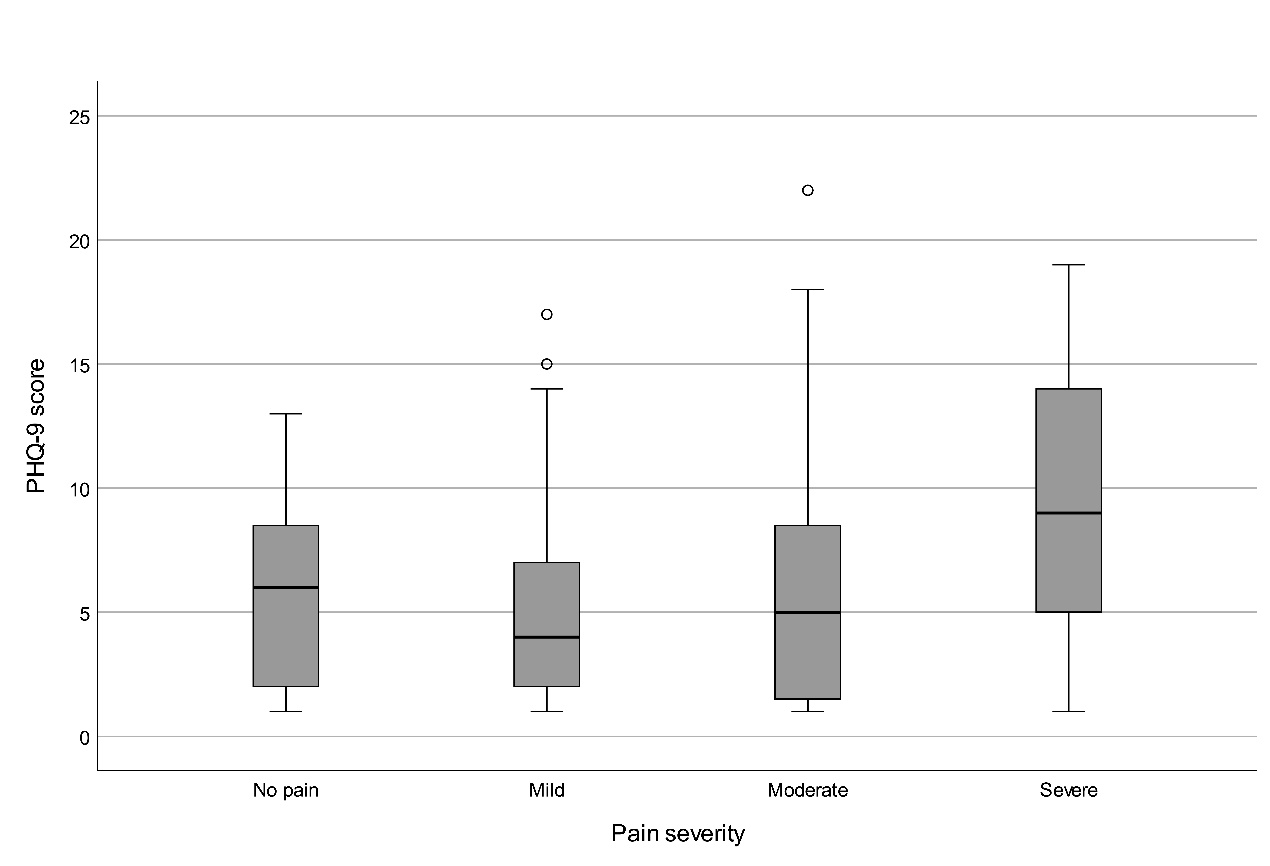


Supplementary Fig. 4. Boxplots of PHQ-9 scores stratified by pain severity levels. Boxes indicate the median and IQR; whiskers extend to 1.5 × IQR; open circles (○) represent outliers (>1.5 × IQR), and asterisks. PHQ-9: Patient Health Questionnaire-9.


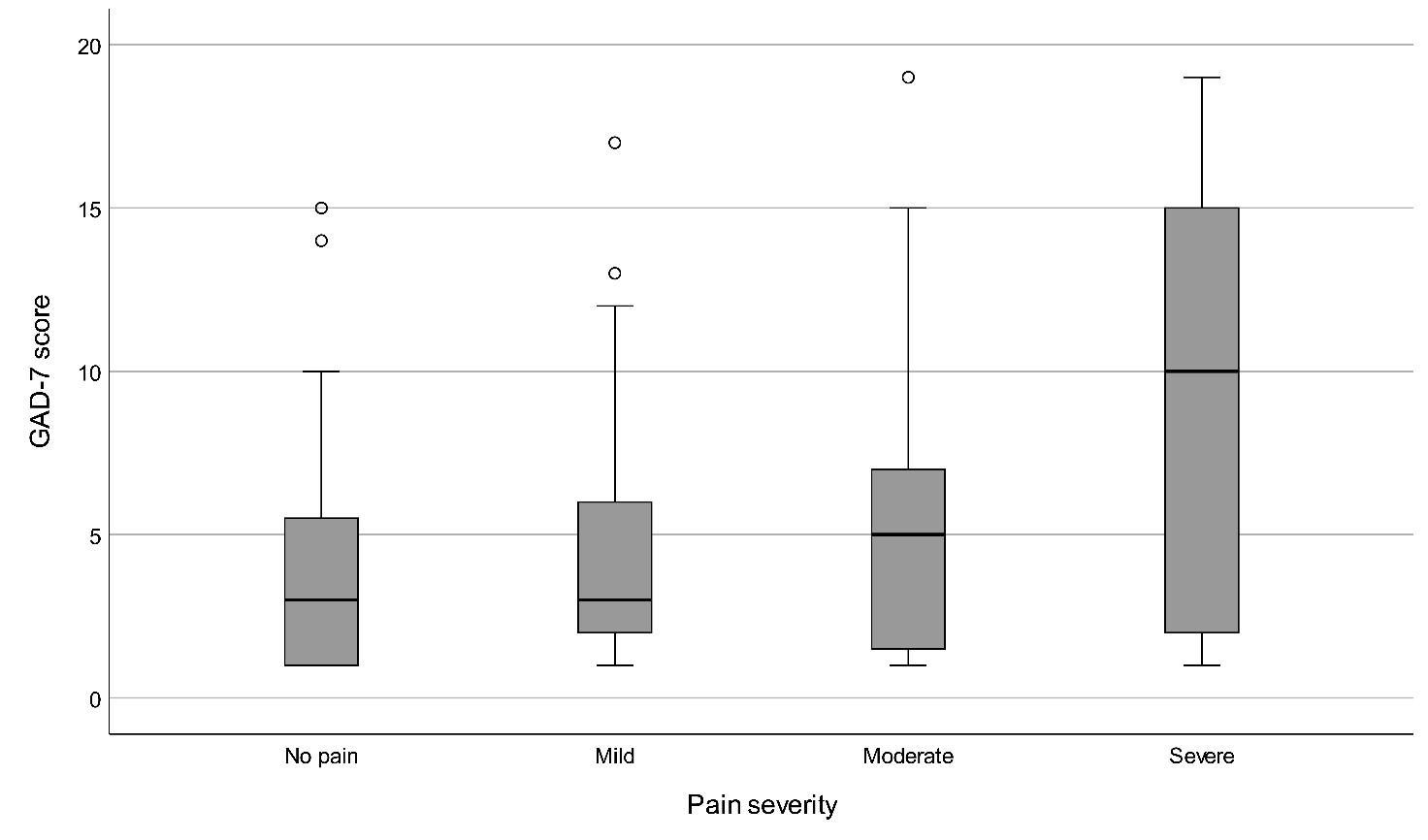


Supplementary Fig. 5. Boxplots of GAD-7 scores stratified by pain severity levels. Boxes indicate the median and IQR; whiskers extend to 1.5 × IQR; open circles (○) represent outliers. GAD-7: Generalized Anxiety Disorder-7.


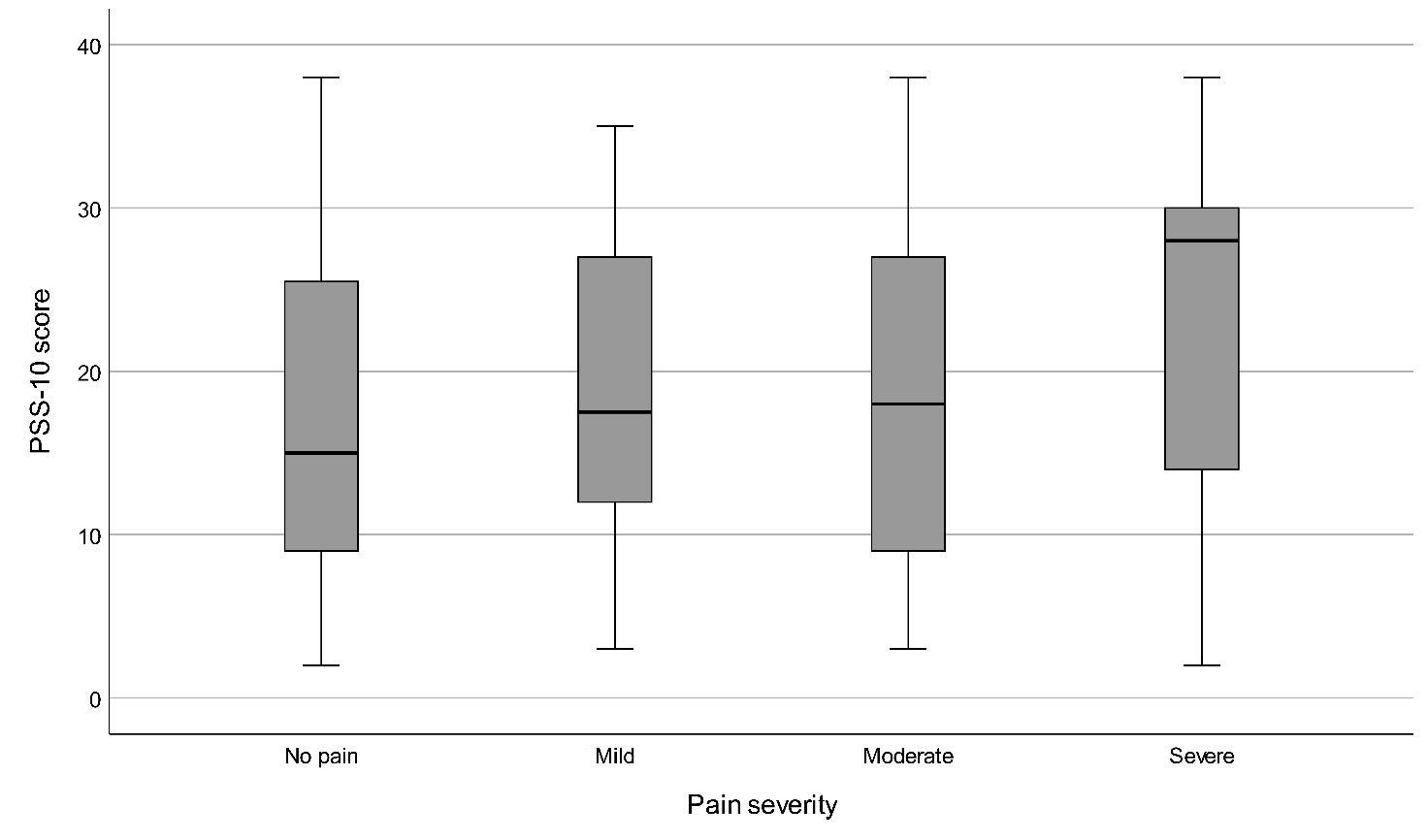


Supplementary Fig. 6. Boxplots of PSS-10 scores stratified by pain severity levels. Boxes indicate the median and IQR; whiskers extend to 1.5 × IQR. PSS-10: 10-item Perceived Stress Scale.


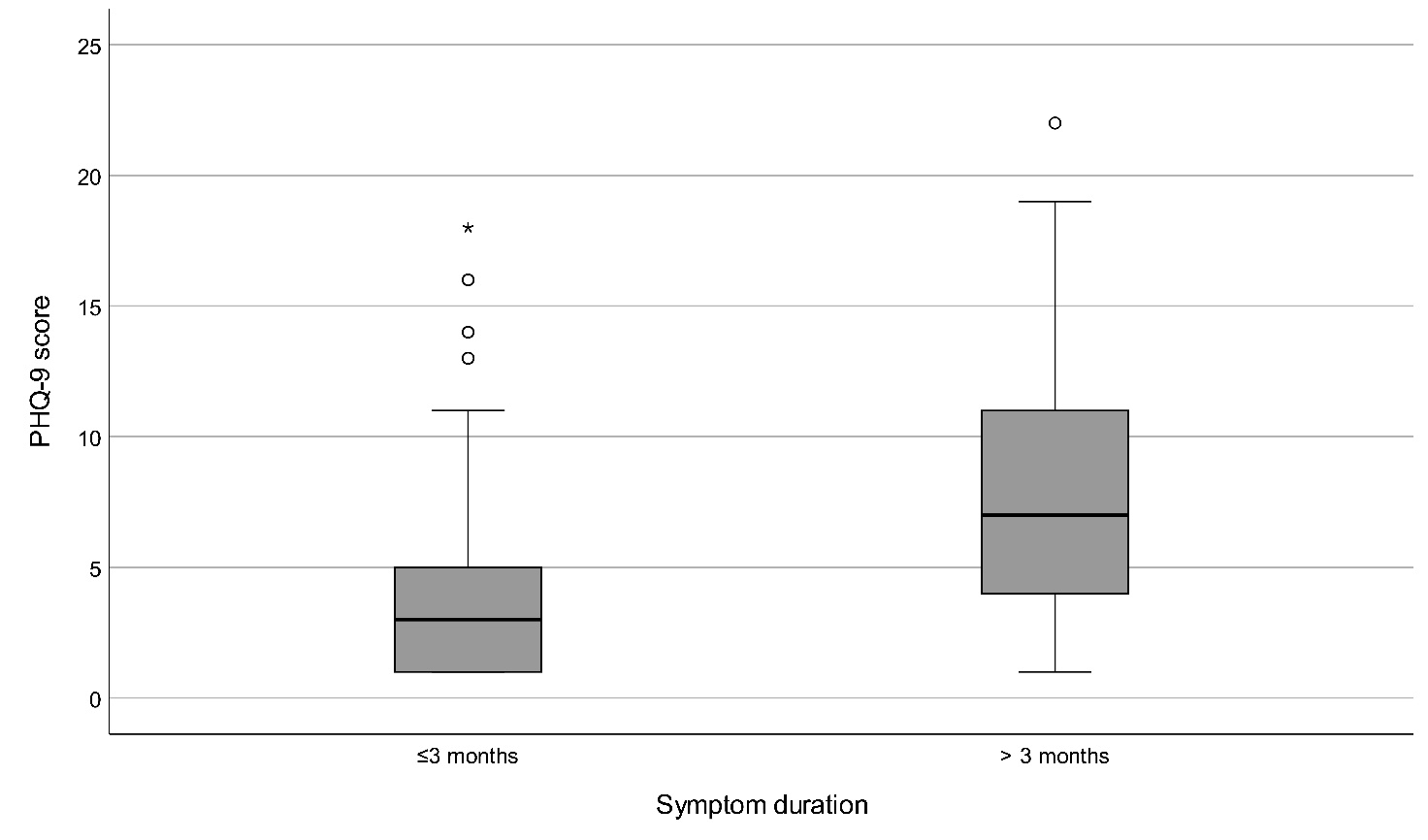


Supplementary Fig. 7. Boxplots of PHQ-9 scores stratified by symptom duration groups. Boxes indicate the median and IQR; whiskers extend to 1.5 × IQR; open circles (○) represent outliers, and asterisks (*) show extreme outliers. PHQ-9: Patient Health Questionnaire-9.


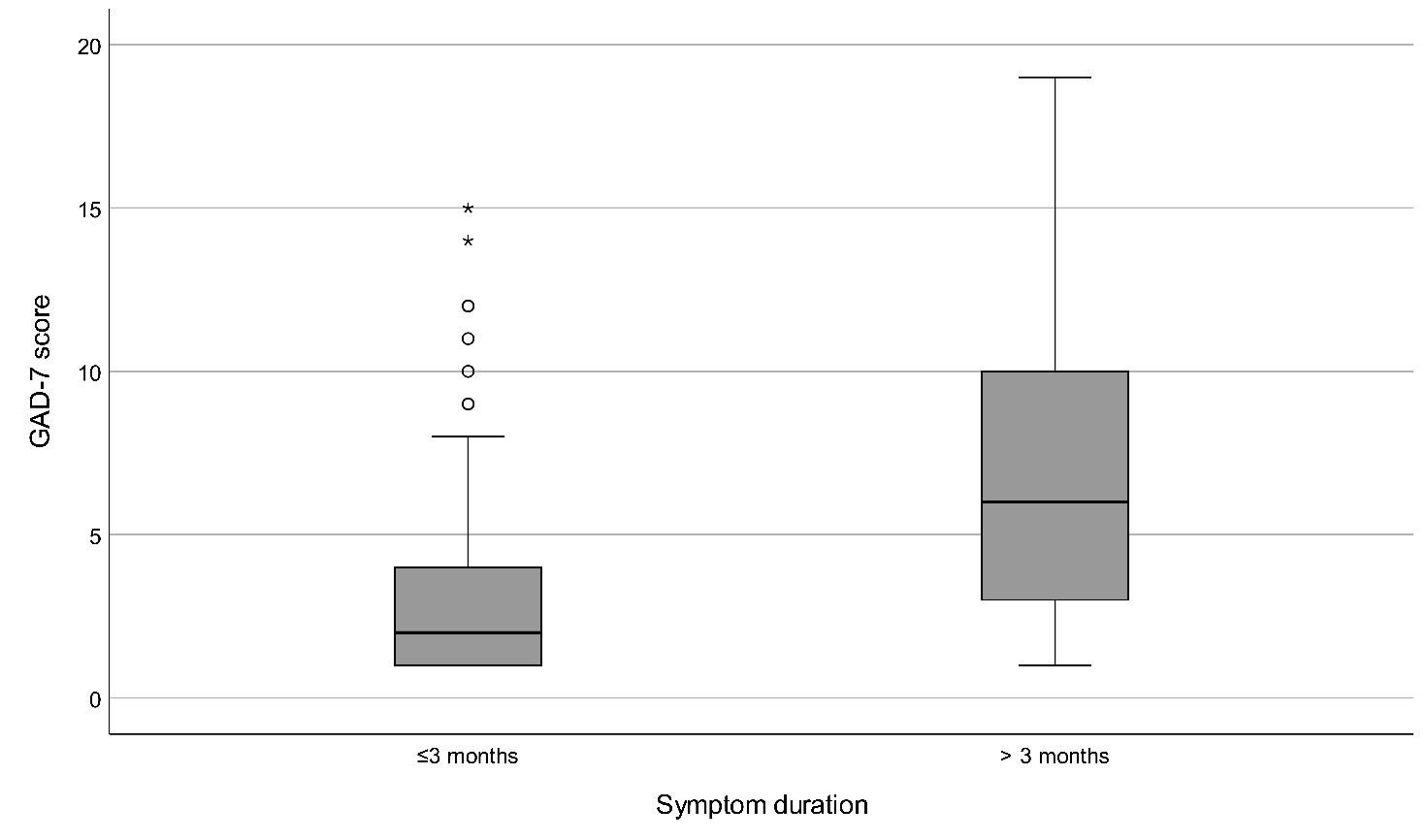


Supplementary Fig. 8. Boxplots of GAD-7 scores stratified by symptom duration groups. Boxes indicate the median and IQR; whiskers extend to 1.5 × IQR; open circles (○) represent outliers, and asterisks (*) show extreme outliers. GAD-7: Generalized Anxiety Disorder-7.


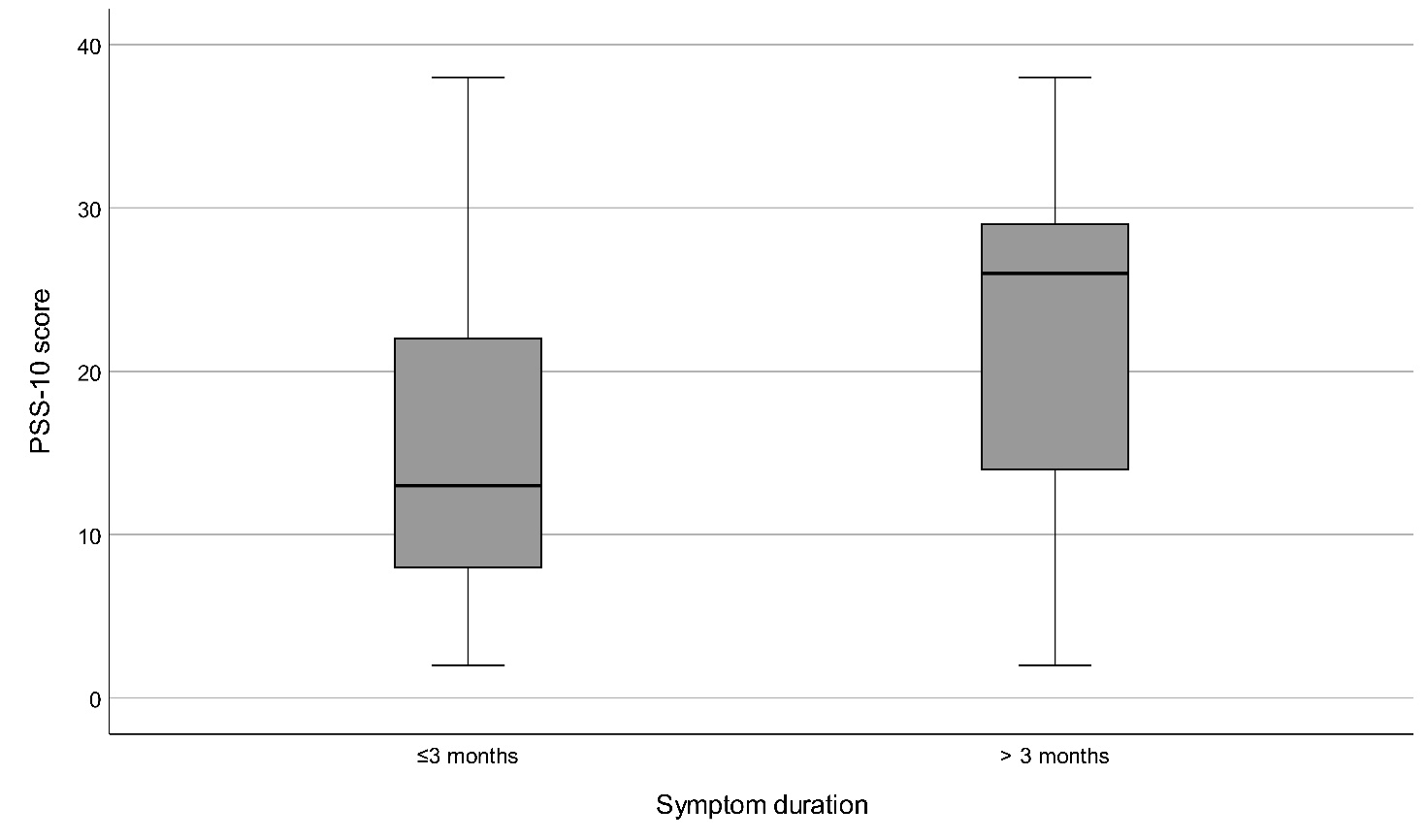


Supplementary Fig. 9. Boxplots of PSS-10 scores stratified by symptom duration groups. Boxes indicate the median and IQR; whiskers extend to 1.5 × IQR. PSS-10: 10-item Perceived Stress Scale.
